# Ultrasensitive Electrochemical Immunoassays of IgG and CA125 Based on Glucose Oxidase-Catalyzed Signal Amplification with Gold Staining

**DOI:** 10.3390/bios15100689

**Published:** 2025-10-11

**Authors:** Long Chao, Zhisong Wu, Shiqiang Qi, Aigui Xu, Zhao Huang, Dexuan Yan

**Affiliations:** 1 Hunan Key Laboratory of Biomedical Nanomaterials and Devices, School of Biological Sicence and Medical Engineering, Hunan University of Technology, Zhuzhou 412007, China; 2 Key Laboratory of Chemical Biology and Traditional Chinese Medicine Research, Ministry of Education of China, College of Chemistry and Chemical Engineering, Hunan Normal University, Changsha 410081, China; 3 College of Design and Engineering, National University of Singapore, 21 Lower Kent Ridge Road, Singapore 119077, Singapore

**Keywords:** enzyme-catalyzed signal amplification, gold staining, low sample volume, ultrasensitive immunoassay

## Abstract

Herein, we propose an ultrasensitive electrochemical immunosensor based on glucose oxidase labeling and enzyme-catalyzed Au staining. In brief, the primary antibody (Ab_1_), bovine serum albumin, an antigen and then a bionanocomposite that contains a second antibody (Ab_2_), poly(3-anilineboronic acid) (PABA), Au nanoparticles (AuNPs) and glucose oxidase (GOx) are modified on a glassy carbon electrode coated with multiwalled carbon nanotubes, yielding a corresponding sandwich-type immunoelectrode. In the presence of glucose, a chemical reduction of NaAuCl_4_ by enzymatically generated H_2_O_2_ can precipitate a lot of gold on the Ab_2_-PABA-AuNPs-GOx immobilized immunoelectrode. In situ anodic stripping voltammetry (ASV) detection of gold in 8 μL 1.0 M aqueous HBr-Br_2_ is conducted for the antigen assay, and the ASV detection process takes approximately 6 min. This method is employed for the assay of human immunoglobulin G (IgG) and human carbohydrate antigen 125 (CA125), which demonstrates exceptional sensitivity, high selectivity and fewer required reagents/samples. The achieved limits of detection (S/N = 3) by the method are 0.25 fg mL^−1^ for IgG (approximately equivalent to containing 1 IgG molecule in the 1 microlitre of the analytical solution) and 0.1 nU mL^−1^ for CA125, which outperforms many previously reported results.

## 1. Introduction

Sensitive, rapid and convenient biochemical analysis is very important for early diagnosis of diseases [1,2,3,4]. As a major disease that is facing humanity, the treatment and accurate diagnosis of cancer are equally important [5,6]. For the detection of relevant small molecules, nucleic acid assays and immunoassays are commonly used and are effective methods for early warnings of cancer [7,8,9]. Among these, an enzyme-labeled and metal-amplified amperometric immunoassay is a promising method for sensitive and rapid analysis [10,11]. For instance, labeled enzyme-catalyzed metal deposition has been used to produce and amplify the amperometric immunoassay signals, because of the sensitive and convenient analysis of metal by anodic stripping voltammetry (ASV) [12,13]. The labeled enzymes include alkaline phosphatase (ALP) [12], horseradish peroxidase (HRP) [14] and glucose oxidase (GOx) [15], and the enzymatically deposited metal mainly involves Ag [16] and Au [17]. Moreover, metal deposition generated by enzymatic catalysis has been used for electrochemical bioassays based on other bioaffinity types, such as nucleic acid hybridization [18,19]. Due to the localized enzyme catalysis, the enzyme-catalyzed metal deposition should have the advantage of efficiently capturing the metal at the labeled enzyme on the immunoelectrode for in situ electrochemical sensing. The ASV analysis of enzyme-labeled and metal-amplified amperometric assays based on a solution-displacement strategy have been reported, but the protocol suffers from the sample solution dilution effect [20].

To our knowledge, enzyme labeling for a sandwich-type immunoassay involves two typical protocols: (1) chemical bonding of an enzyme molecule to an antibody 2 (Ab_2_) molecule; and (2) immobilizing Ab_2_ molecules and enzyme molecules on/in an appropriate matrix, such as polymers and/or nanomaterials [12,21]. Obviously, the matrix-based protocol has the advantage of labeling more enzyme molecules for enhanced signaling. A polymeric bionanocomposite is composed of nanomaterials, biomacromolecules and probably more components that are entrapped into the polymer networks, which is multifunctional, robust, cost-effective, and convenient in preparation, and has found wide applications in many fields including biosensing [22,23]. The poly(3-anilineboronic acid) (PABA) have been demonstrated to be able to easily entrap GOx at high load/activity through the glycosyl–boronic group interaction and encapsulate/adsorb Au nanoparticles (AuNPs) for an amperometric enzymatic assay of glucose [24]. Up to now, there have been very few reports about the performance improvement of electrochemical immunoassays based on the simultaneous embedding of an enzyme and an antibody with PABA. We thus believe that the Ab_2_-PABA-AuNPs-GOx bionanocomposite (matrix-based protocol) should be an ideal material that is worthy of exploiting for a sandwich-type amperometric immunoassay and also has the following advantages: (1) the loading capacity of highly active enzymes can be increased in the PABA with high biocompatibility, which can significantly enhance the metal signals generated by the enzyme-catalyzed reaction; and (2) the AuNPs in the Ab_2_-PABA-AuNPs-GOx bionanocomposite may act as a “seed”, inducing the more rapid deposition and growth of gold generated by the enzyme-catalyzed reaction, further enhancing the amplification effect of the metal signal.

Here, we report an ultrasensitive in situ microliter-droplet anodic stripping voltammetry for immunoassays of IgG and CA125, which exploited the secondary antibody-PABA-AuNPs-glucose oxidase (Ab_2_-PABA-AuNPs-GOx) bionanocomposite as a new carrier of the second antibody on the basis of GOx-catalyzed Au staining.

## 2. Materials and Methods

### 2.1. Instruments and Reagents

A CHI660E electrochemical workstation was used for all electrochemical experiments here. A glassy carbon electrode (GCE) with a 3.0 mm diameter was used as the working electrode (WE) and a tiny graphite rod as the counter electrode (CE). A KCl-saturated calomel electrode (SCE) with a small-sized capillary filled with saturated KNO_3_ was used as reference electrode (RE). All potentials are reported here, versus SCE. The quartz crystal microbalance (QCM) technique was used to investigate the process of the immunoassay here, and QCM experiments were carried out on an HP4395A impedance analyzer [25]. AT-cut 9 MHz piezoelectric quartz crystals with a 12.5 mm wafer diameter and coated with a gold film of a 6.0 mm diameter on one side were used (QCM Au, Beijing Chenjing Electronics Co., Ltd, Beijing, China), and the side of a gold-plated surface soaked in the solution and the other side faced the air. Ultraviolet-visible (UV-Vis) spectra were collected on a UV-2450 spectrophotometer (Shimazu Co., Kyoto, Japan). Scanning electron microscopy (SEM) images and energy-dispersive X-ray (EDX) spectra were collected on a field emission scanning electron microscope, equipped with an energy-dispersive X-ray spectrometer.

Goat anti-human IgG (anti-IgG) and IgG (MW = 150 kD) were provided by Beijing Dingguo Changsheng Biotechnology Co., Ltd. (Beijing, China). Mouse monoclonal anti-human CA125 (Ab_1_), anti-CA125 (Ab_2_) and CA125 (MW = 200 kD) were obtained from Beijing Key Biotech. Co. Ltd. Note that anti-IgG was used both as Ab_1_ and Ab_2_ here, in the process of electrode modification and immunoassay for convenience, as previously reported [26]. GOx (EC 1.1.3.4; type II from Aspergillus niger, activity ≈ 150 kU g^−1^), bovine serum albumin (BSA) and 3-aminophenylboronic acid (ABA) monohydrate were purchased from Sigma. HAuCl_4_, glucose, hydrobromic acid (≥40%), 1-ethyl-3-(3-dimethyllaminopropyl) carbodiimide hydrochloride (EDC) and N-hydroxysuccinimide (NHS) were supplied by Sinopharm Chemical Reagent Co., Ltd. Multiwalled carbon nanotubes (MWCNTs, purity 96%, average diameter of 30 nm, and average length of 10 μm) were supplied by Chengdu Organic Chemistry Co., LTD (Chengdu, China). The washing and blocking buffer solution for the immunoassay was 0.01 M Phosphate-Buffer Solution (PBS, pH 7.4, contain 10 mM NaH_2_PO_4_−Na_2_HPO_4_ + 0.15 M NaCl). The substrate solution for the enzymatic reaction containing 5 mM glucose and 2 mM NaAuCl_4_ was freshly prepared every day. The clinical serum samples were donated by the Second Xiangya Hospital of Central South University (Changsha, China) and the content of CA125 was detected by chemiluminescence on a fully automatic biochemical analyzer in the hospital for each sample. The acquisition of the samples was approved by the hospital’s ethics committee and was permitted for research purposes. All the blood samples used here were obtained from the remaining samples that were only used for routine clinical tests and were completely anonymized.

### 2.2. Preparation of Ab_2_-PABA-AuNPs-GOx

**Figure 1:**
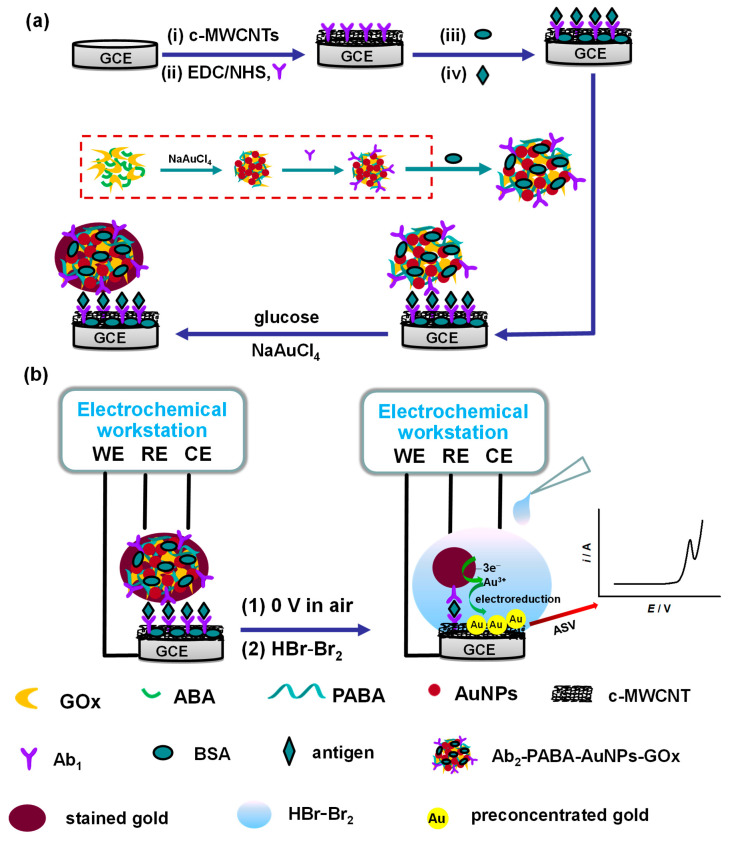
Principle scheme of electrode fabrication (**a**) and immunoassay procedure (**b**) in our electrochemical method.

The preparation of Ab_2_-PABA-AuNPs-GOx is shown in Figure 1a and details are as follows. The GOx–ABA composite containing 10 mg GOx and 5 mg ABA in 1 mL PBS was shaken for 25 min to achieve a reaction equilibrium and 400 μL 19.8 mM, then NaAuCl_4_ was added for a 40 min reaction. In the aforementioned mixed solution, the ABA was oxidatively polymerized by NaAuCl_4_ to form PABA; meanwhile, the Au(III) in NaAuCl_4_ reduced into Au(0) and formed AuNPs. Afterward, centrifugation at 13,000 rpm for 10 min was conducted, the suspension was removed, and the precipitate was washed with PBS. After another centrifugation for supernatant removal, the GOx-PABA-AuNPs precipitate was redispersed into 1 mL of 0.01 M pH 7.4 PBS containing 10 μg Ab_2_ and mixed gently at 4 °C overnight. In order to block the nonspecific sites, the resulting Ab_2_-PABA-AuNPs-GOx was redispersed into 0.5 mL of PBS with a pH of 7.4, containing 1.0% (*w/v*) BSA. After centrifuging at 13,000 rpm for 10 min twice, it was stored in the refrigerator at 4 °C when not in use. Here, ABA can be used as a functional reagent to connect the glycosyl of GOx, and its aniline moiety and excess ABA can react with NaAuCl_4_ to generate the bionanocomposite of AuNPs, PABA and GOx in a one-pot method. The redundant boronic acid groups can be linked to the glycosyl of Ab_2,_ and the AuNPs can also combine Ab_2_ with Au-S bonding.

For comparison, the Ab_2_-AuNPs-GOx bionanocomposite was also synthesized and used as a carrier of the second antibody for ASV immunoassays of IgG and CA125. Here, the AuNPs were synthesized in advance by the traditional method of reducing HAuCl_4_ with sodium citrate. Briefly, 100 mL of 0.01% (*w*/*v*) aqueous HAuCl_4_ was brought to the boiling point under vigorous stirring, and 2.5 mL of 1% (*w*/*v*) aqueous trisodium citrate was quickly added to the boiling solution. When the solution turned deep red in color, boiling was pursued for an additional 2 min, and this adjusted the pH value of the AuNPs dispersion to 7.4. Next, 10 mg GOx was added in 1 mL of the AuNPs dispersion, and also shaken for 25 min to achieve reaction equilibrium. The subsequent synthesis steps are similar to those for synthesizing Ab_2_-PABA-AuNPs-Gox, as mentioned above.

### 2.3. Construction of Immunoelectrodes

The GCE was carefully polished using Al_2_O_3_ powder (particle size 0.5 μm and 0.05 μm), and then thoroughly rinsed with water. To remove any residual Al_2_O_3_ particles, the polished electrode was ultrasonically cleaned sequentially in water, then ethanol, and again in water. Subsequently, the GCE was treated with concentrated H_2_SO_4_ for 10 s. Finally, electrochemical cleaning was performed by cyclic voltammetry (CV, potential range: −1.0 to 1.0 V, scan rate: 100 mV s^−1^) in 0.10 M aqueous H_2_SO_4_, until stable and reproducible voltammograms were obtained.

Firstly, the primal MWCNTs underwent carboxylation by being treated with a 3:1 (*v/v*) mixture of H_2_SO_4_/HNO_3_ under vigorous sonication for 3 h. The resulting product was then repeatedly rinsed with ultrapure water until the wash solution reached neutrality. As illustrated in Figure 1a, 4 µL of the carboxylated MWCNTs solution (0.5 mg mL^−1^) was drop-coated onto a cleaned GCE and air-dried in a ventilated environment to form the MWCNTs-modified GCE (MWCNTs/GCE). Subsequently, the MWCNTs/GCE was immersed in 100 μL of an activation solution, containing 600 mM EDC and 125 mM NHS, for 20 min to activate the carboxyl groups. The electrode was then incubated overnight at 4 °C with 7 μL of a 1 mg mL^−1^ Ab_1_ solution in PBS. After washing away unbound Ab_1_ with PBS, 6.0 µL of PBS containing 5% BSA was dropped on the surface of Ab_1_/MWCNTs/GCE to block nonspecific binding sites for 1.5 h, yielding the BSA/Ab_1_/MWCNTs/GCE. Following another PBS rinse, the modified electrode was stored in PBS at 4 °C when not in use.

The antigen assay procedure is illustrated in Figure 1b. The prepared electrode was firstly incubated in 8 μL PBS containing a certain concentration antigen at 37 °C for 1 h to yield antigen/BSA/Ab_1_/MWCNTs/GCE. After PBS rinsing, the electrode was further incubated in 8 μL of PBS with Ab_2_-PABA-AuNPs-GOx at 37 °C for 50 min, resulting in Ab_2_-PABA-AuNPs-GOx/antigen/BSA/Ab_1_/MWCNTs/GCE. The electrode was then thoroughly rinsed with PBS to remove the non-specific adsorbate. Next, 10 μL of a solution containing 2 mM NaAuCl_4_ and 5 mM glucose was applied to the modified electrode. Following a 2 h enzymatic catalytic reaction at 37 °C, a gold/Ab_2_-PABA-AuNPs-GOx/antigen/BSA/Ab_1_/MWCNTs/GCE structure was formed. Finally, the electrode was rinsed three times with ultrapure water and stored under dry conditions until use.

### 2.4. ASV Detection Process

An immunoassay of anodic stripping voltammetry (ASV) based on metal signal is as described below (as shown in Figure 1b). Firstly, the Au loaded on Ab_2_-PABA-AuNPs-GOx bionanocomposite were dissolved by acidic HBr into Au(III), then Au(III) diffused onto the electrode surface, and finally the Au(III) is electroreduced to Au(0) under the condition of applying a reduction potential. Therefore, we firstly applied a cathodic potential of 0 V to the immunoelectrode, and the potential was sufficiently negative, which ensured the electrodeposition of gold in the reaction solution. Next, the enzyme-catalyzed gold was dissolved by adding 10 μL of 1.0 M aqueous HBr-Br_2_, and the electroreduction of dissolved gold on the surface of the electrode was simultaneously started at 0 V and lasted for 350 s. Here, it has been reported that the effect of acidic HBr-Br_2_ on gold extraction is better than that of traditional cyanide reagents [27], and Br_2_ is necessary for gold dissolution in 1.0 M of HBr medium, according to the report [28]. At the last 30 s of the electroreduction of dissolved gold, 3 μL of 1.0 M HBr solution containing 4 mM 3-phenoxypropionic was added to the surface of the electrode. Usually, the effect of electroreduction of gold cations will decrease due to the reoxidation of electrodeposited gold above 0.7 V in the presence of Br^−^/Br_2_. Therefore, before ASV recording, the superfluous bromine was consumed by adding a small amount of 3-phenoxypropionic acid here, which was a reaction reagent of bromine. Finally, linear sweep voltammetry (scanning from 0 to 1.0 V at a rate of 100 mV s^−1^) was utilized to obtain the ASV current responses of the electrodeposited gold.

## 3. Results and Discussion

### 3.1. Characterization of Ab_2_-PABA-AuNPs-GOx and the Immunoelectrode

**Figure 2:**
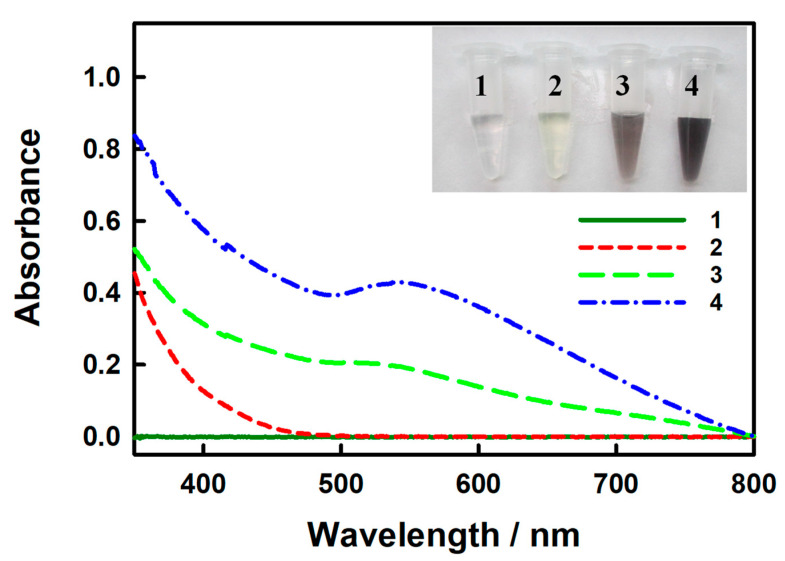
UV-Vis spectra and digital picture (Insert) of 0.50 mL PBS containing 5 mM glucose (1), 0.50 mL PBS containing 5 mM glucose and 2 mM NaAuCl_4_ (2), 0.50 mL PBS containing only 200-fold diluted as-prepared BSA/Ab_2_-PABA-AuNPs-GOx suspension, (3) and 0.50 mL PBS containing 200-fold diluted as-prepared BSA/Ab_2_-PABA-AuNPs-GOx suspension, 5 mM glucose and 2 mM NaAuCl_4_ after its enzyme-catalyzed reaction for 2 h (4).

Here, we employed GOx to label Ab_2_ for enzyme-catalyzed gold-deposition. We monitored the color change in the enzyme-catalyzed reaction to track the reaction process by capturing pictures and recording UV-Vis absorption spectra, as shown in Figure 2, and the insert of Figure 2 shows the visual inspection results. Initially, the glucose solution was colorless and exhibited no absorption peak (curve 1). Upon adding 2 mM NaAuCl_4_, the solution turned faint yellow, but there was still no obvious absorption peak (curve 2). The color of the BSA/Ab_2_-PABA-AuNPs-GOx suspension was dark brown with a weak absorption peak at 515 nm (curve 3). After addition of the enzyme-catalyzed solution (5 mM glucose and 2 mM NaAuCl_4_, the final concentration) and reaction for 2 h, the BSA/Ab_2_-PABA-AuNPs-GOx suspension darkened further, and the absorption peak appeared at ca. 544 nm for AuNPs (curve 4). We also investigated the situation of gold staining when there are no AuNPs as seeds, shown in Figure S1. After the reaction of 2 mM NaAuCl_4_ and 50 µM H_2_O_2_ (used to replace the enzyme-generated H_2_O_2_ here) for 2 h without the presence of AuNPs, the absorption peak that appeared at ca. 544 nm for AuNPs is lower than that of curve 4, and we believe that the AuNPs can accelerate the gold staining.

**Figure 3:**
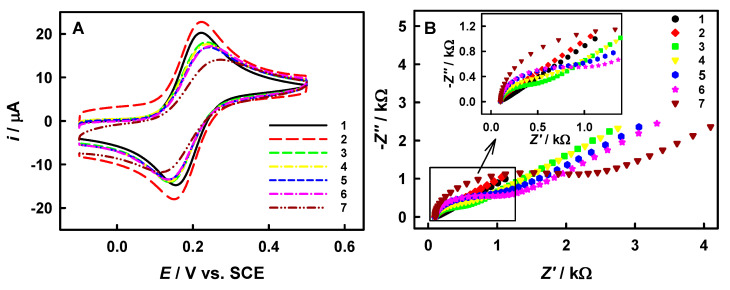
Cyclic voltammogram (**A**) and EIS (**B**) of bare GCE (1), MWCNTs/GCE (2), Ab_1_/MWCNTs/GCE (3), BSA/Ab_1_/MWCNTs/GCE (4), antigen/BSA/Ab_1_/MWCNTs/GCE (5), Ab_2_-PABA-AuNPs-GOx/antigen/BSA/Ab_1_/MWCNTs/GCE (6), and gold/Ab_2_-PABA-AuNPs-GOx/antigen/BSA/Ab_1_/MWCNTs/GCE (7) in 0.1 M PBS containing 2.0 mM K_4_Fe(CN)_6_. The inset in panel B is an enlarged image within the box. The scan rate of CV: 50 mV s^−1^, frequency range of EIS: 100 kHz~1 Hz, 0.21 V vs. SCE.

Taking the IgG immunoassay as an example, the modifications of the electrode by our method were investigated by CV and electrochemical impedance spectroscopy (EIS), as shown in Figure 3. A potential of 0.21 V vs. SCE (the formal potential of Fe(CN)_6_^3−^/^4−^ redox pair) was applied to the WE. Here, Ab_1_, antigen and Ab_2_ correspond to anti-IgG, IgG and anti-IgG, respectively. As the modifications of electrode occur step-by-step, the potential difference in anodic and cathodic peaks of the Fe(CN)_6_^3−/4−^ probe has gradually become larger, as well as the diameter of the EIS semicircle. The CV and EIS results show a sequential decrease in the electrochemical reversibility of the Fe(CN)_6_^3−^/^4−^ redox probe across modified electrodes, following this order: bare GCE ≈ MWCNTs/GCE > Ab_1_/MWCNTs/GCE > BSA/Ab_1_/MWCNTs/GCE > antigen/BSA/Ab_1_/MWCNTs/GCE > Ab_2_-PABA-AuNPs-GOx/antigen/BSA/Ab_1_/MWCNTs/GCE > gold/Ab_2_-PABA-AuNPs-GOx/antigen/BSA/Ab_1_/MWCNTs/GCE. It demonstrates that the modification of non-conducting biomacromolecule/bionanocomposite can decrease the electrode activity. Here, it is worth mentioning that the Ab_2_-PABA-AuNPs-GOx and stained gold cannot increase the electrode activity because the polymeric bionanocomposite was entrapped within the AuNPs, and then the stained gold was far away from the electrode surface.

Furthermore, the IgG immunoassay was also investigated by the QCM technique here, as shown in Figure S2. Firstly, a clean QCM Au was soaked in 0.01 M of stirred PBS. Then, after the addition of Ab_1_ to a final concentration of 0.05 mg mL^−1^ into the stirred PBS solution, followed by incubation for nearly 1 h, a frequency decrease of 285 Hz was observed, (curve 1), signifying successful Ab_1_ adsorption on QCM Au. Surplus Ab_1_ were washed away with the 0.01 M PBS. The nonspecific binding sites were blocked by subsequently immersing QCM Au adhered with Ab_1_ in a new 0.01 M of stirred PBS, containing 3% BSA for about 45 min, and the QCM exhibited a frequency decrease of about 27 Hz (curve 2). After the QCM Au was washed with 0.01 M PBS, an antigen at a final concentration of 0.05 mg mL^−1^ was added into fresh PBS under stirring conditions. This resulted in a frequency decrease of 241 Hz (curve 3), meaning the immunoadsorption reaction between the Ab_1_ and the antigen had successfully occurred and immunoprecipitated on the surface of QCM Au. Followed by washing the QCM Au with PBS again, a solution containing 0.05 mg mL^−1^ Ab_2_-PABA-AuNPs-GOx (final concentration) was injected into a new stirred PBS, causing an additional frequency drop of approximately 93 Hz (curve 4), which indicates that the sandwich-like immune structure has successful formed on the surface of QCM Au. Finally, after another washing with PBS, the QCM Au was soaked in a new stirred 0.01 M PBS, then 5 mM glucose and 2 mM NaAuCl_4_ (final concentrations) were added into the stirred PBS solution, a QCM decreasing frequency of 2 kHz was observed (curve 5), which indicated that the enzyme-catalyzed gold deposition had successfully formed on the QCM Au.

**Figure 4:**
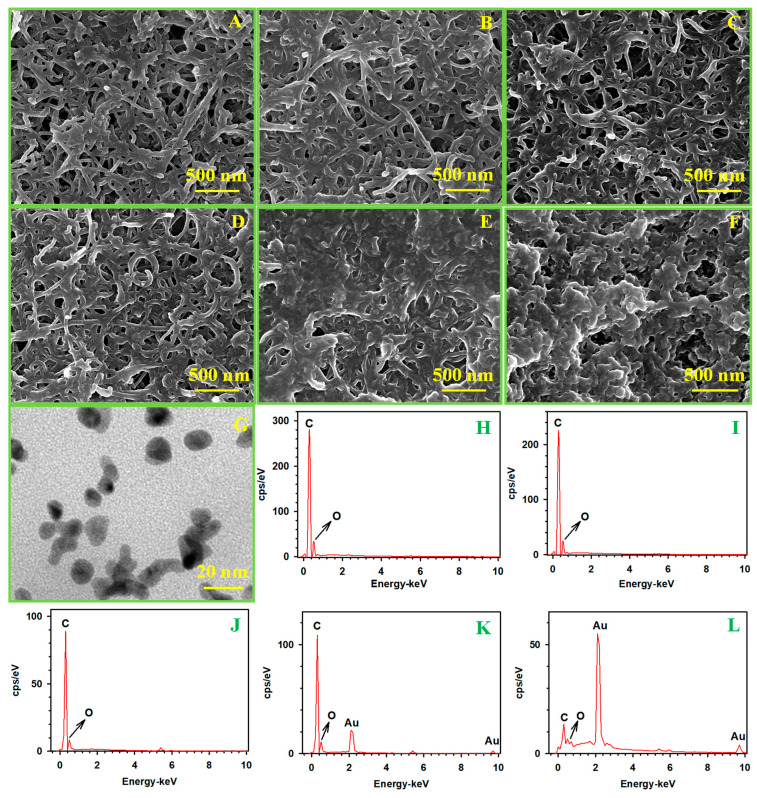
SEM images, EDX spectra of BSA/Ab_1_/MWCNTs/GCE (**A**,**H**), antigen/BSA/Ab_1_/MWCNTs/GCE (**B**,**C**,**J**), Ab_2_-PABA-AuNPs-GOx/antigen/BSA/Ab_1_/MWCNTs/GCE (**D**,**E**,**I**,**K**), gold/Ab_2_-PABA-AuNPs-GOx/antigen/BSA/Ab_1_/MWCNTs/GCE (**F**,**K**) and TEM image of Ab_2_-PABA-AuNPs-GOx (**G**). Concentration of IgG: 0 ng mL^−1^ (**B**,**D**,**H**) and 500 ng mL^−1^ (**C**,**E**,**F**,**J**,**L**).

The fabrication of the IgG immunoelectrode was further characterized using SEM and EDX, with results presented in Figure 4. On the BSA/Ab_1_/MWCNT/GCE and antigen/BSA/Ab_1_/MWCNTs/GCE, nanotubes were seen, and no obvious morphological changes were observed (Figure 4A,C), probably because the protein layer was thin. On the Ab_2_-PABA-AuNPs-GOx/antigen/BSA/Ab_1_/MWCNT/GCE, the morphology of the immunoelectrode had obviously changed, proving the successful immune binding of Ab_2_-PABA-AuNPs-GOx to the antigen (Figure 4E). After the enzyme-catalyzed gold-staining reaction, the Ab_2_-PABA-AuNPs-GOx became larger, supporting that gold staining occurs only on Ab_2_-PABA-AuNPs-GOx. The EDX spectroscopy results for Ab_2_-PABA-AuNPs-GOx/antigen/BSA/Ab_1_/GCE (Figure 4J) and gold/Ab_2_-PABA-AuNPs-GOx/antigen/BSA/Ab_1_/GCE (Figure 4K) demonstrate an increase in both mass and Au content following the enzyme-catalyzed reaction. Compared with BSA/Ab_1_/MWCNT/GCE, the morphology of antigen/BSA/Ab_1_/MWCNTs/GCE and Ab_2_-PABA-AuNPs-GOx/antigen/BSA/Ab_1_/MWCNT/GCE did not change significantly when the antigen concentration was 0 ng mL^−1^ (Figure 4B,D), and the EDX spectroscopy results also indicated that there was no gold on the Ab_2_-PABA-AuNPs-GOx/antigen/BSA/Ab_1_/MWCNT/GCE (Figure 4H). All these experiments have demonstrated the successful implementation of glucose oxidase-mediated gold deposition on functionalized electrodes. Moreover, a TEM image of PABA-AuNPs-GOx was provided, as shown in Figure S3, and the size of PABA-AuNPs-GOx is ca. 10–15 nm. Finally, the successful synthesis of Ab_2_-PABA-AuNPs-GOx was also confirmed by transmission electron microscopy (TEM). Coupled with EDX, the size of Ab_2_-PABA-AuNPs-GOx is ca. 15–20 nm (Figure 4G) and the results of EDX show the presence of gold (shown in Figure S4).

### 3.2. Electrochemical Immunoassay for IgG and CA125

The amount of deposited gold depended on the amount of hydrogen peroxide generated by the enzymatic transformation of the glucose substrate. Thus, the effect of the glucose concentration was assessed first. After gold deposition for 1 h in pH 7.4 PBS containing 2.0 mM NaAuCl_4_ and different concentrations of glucose, the anode oxidation peak current of electrodeposited Au increased significantly, with the glucose concentration up to 5.0 mM, and then the growth of peak current began to level off (as shown in Figure S5A). This result showed that 5.0 mM glucose should correspond to the maximum GOx activity, which was enough for the GOx-catalyzed gold deposition here. Hence, a glucose concentration of 5.0 mM was chosen in the subsequent research.

Moreover, the concentration of gold cation should also be very important for the electrochemical anodic stripping signal of gold. Next, we optimized the concentration of NaAuCl_4_. As shown in Figure S5B, as the concentration of NaAuCl_4_ increases, the anodic stripping peak current increases at a deposition time of Au of 1 h in PBS containing 5.0 mM glucose and a different concentration of NaAuCl_4_. Then, the growth of the peak current tends to reach saturation at 2.0 mM NaAuCl_4_, which was selected as the subsequent experimental condition.

The amount of deposited gold on the surface of the electrode is also obviously related to the enzymatic reaction time. Hence, the effect of the enzymatic reaction time was investigated. It can be observed that the anodic stripping peak current increased rapidly with the enzymatic reaction time until 1 h at the glucose and NaAuCl_4_ concentrations of 5.0 and 2.0 mM, respectively (as shown in Figure S5C). Therefore, we used the enzymatic reaction time of 2 h in the subsequent experiments.

For comparison, the Ab_2_-AuNPs-GOx bionanocomposite was also used as a carrier of the second antibody for the ASV immunoassay of IgG and CA125. The synthesis conditions for Ab_2_-AuNPs-GOx are mostly the same as those optimized for the synthesis of Ab_2_-PABA-AuNPs-Gox, mentioned above. As shown in Figure S6, the ASV signals of Ab_2_-PABA-AuNPs-GOx are significantly higher than that of Ab_2_-AuNPs-GOx for both the immunoassay of IgG (50 ng mL^−1^) and CA125 (50 mU mL^−1^) under the same conditions. The result indicates that the Ab_2_-PABA-AuNPs-GOx has the effect of enhancing the signal on a sandwich-type amperometric immunoassay compared with Ab_2_-AuNPs-GOx, which should be attributed to the enzymes carried by Ab_2_-PABA-AuNPs-GOx, and it generated a large amount of enzyme-catalyzed gold, thereby enhancing the metal signal of ASV.

**Figure 5:**
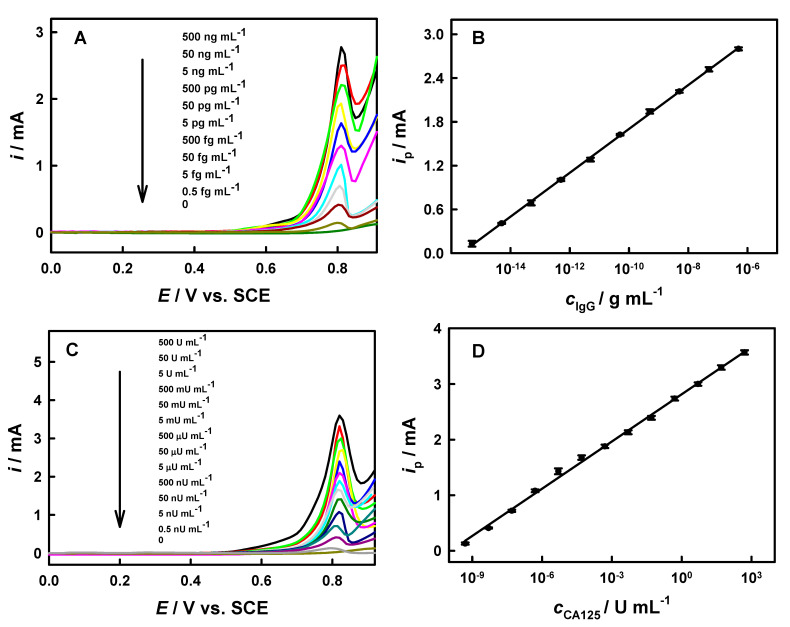
Linear sweep ASV curves for IgG (**A**) or CA125 (**C**) detection, along with their respective calibration curves (**B**,**D**) (n = 3).

Under the optimum conditions, our method demonstrates a linear relationship between the ASV peak current and the logarithm of IgG concentration across a range from 0.5 fg mL^−1^ to 500 ng mL^−1^, achieving a sensitivity of 305 μA dec^−1^ and a LOD of 0.25 fg mL^−1^ (S/N = 3), as shown in Figure 5A,B. According to Avogadro’s constant (6.02 × 10^23^) and the average relative molecular weight of IgG (150 kD), it can be calculated that there is approximately 1 IgG molecule in each microliter of the sample (0.25 × 10^−15^ × 6.02 × 10^23^ × 10^−3^/1.5 × 10^5^ = 1.003 ≈ 1.0) based on the LOD above. The single-molecule-level LOD of IgG obtained through our method here is superior to that reported in most of the previous literature (Table 1), and we think that the electrochemical quantitative detection at the single-molecule level also has great prospects for application in other fields, such as clinical diagnosis and biochemical analysis.

In medicine, the CA125 biomarker is commonly regarded as an important indicator for the diagnosis and management of primary invasive epithelial ovarian/tubal cancer (iEOC). iEOC is usually regional and has no obvious symptoms at early stages, whereas the metastasis of cancer cells to other parts of the body may cause pain, especially spreading to the bones [29]. Therefore, the early diagnosis of iEOC is very important for successful treatment and curative surgery. Similarly, the electrochemical immunoassay of CA125 was conducted here.

The ASV peak current demonstrates excellent linearity with the CA125 concentration across a wide range (0.5 nU mL^−1^ to 500 U mL^−1^) under ideal conditions, showing 283 μA dec^−1^ sensitivity and a detection limit of 0.1 nU mL^−1^ (S/N = 3; Figure 5C,D). The LOD of CA125 by our method surpasses most of the reported values (Table 1).

**Table 1:** Some performance comparisons of the immunoassay for IgG and CA125.

Analyte	Label	Analytical Technique	LDR /ng mL^−1^ for IgG and U mL^−1^ for CA125	LOD /pg mL^−1^ for IgG and U mL^−1^ for CA125	References
IgG	HRP	Chronoamperometry for H_2_O_2_	0.1–200	50	[30]
	glucose oxidase	Chronoamperometry	0.005–1.0	2	[26]
	[Fe(CN)_6_]^3−/4−^ probe	Differential pulse voltammetry	0.1–1.0 × 10^4^	32	[31]
	AuNPs and ALP	ASV for catalytically deposited Ag	0.01–250	4.8/6.1	[12]
	CdTe QDs	Fluorometry/square wave voltammetry	0.1–500 /5 × 10^−3^–100	30/5	[32]
	HRP	Potentiostatic amperometry	0.33–75	0.1	[33]
	AuNPs	ASV for silver staining	4 × 10^−7^–400	0.2 × 10^−3^	[34]
	AuNPs	ASV for copper staining	4 × 10^−7^–400	0.3 × 10^−3^	[35]
	glucose oxidase	ASV for enzyme-catalyzed deposited Au	5 × 10^−8^–500	0.25 × 10^−3^	This work
CA125	CdS/SnS_2_	Photoelectrochemical	10^−7^–10^2^	5.48 × 10^−8^	[36]
	Au@Pd	Electrochemical	0.002–20	0.001	[37]
	HRP	Differential pulse voltammetry	0.1–300	0.027	[38]
	Au-Ag core shell	Electrochemical impedance spectroscopy	1–150	1.03	[39]
	Au/Ru	Resonance Rayleigh scattering	1.3–80	0.6	[40]
	ALP	Optical/Electrochemical	5–1000/5–1000	1.3/40	[41]
	SiO_2_(thionine-HRP)	Electrochemical immunoassay	0.1–450	0.1	[42]
	CdSe	Electrochemiluminescence	1.0 × 10^−4^–0.3	5.8 × 10^−5^	[43]
	glucose oxidase	ASV for catalytically deposited Au	5 × 10^−10^–500	10 × 10^−10^	This work

We also evaluated the practicability for a CA125 assay with our method in five clinical human serum samples. Our results are basically consistent with the results from the hospital, conducted on a fully automatic biochemical analyzer by a chemiluminescence assay (within ±10% RSD), as listed in Table 2, demonstrating the clinical applicability of our method for disease biomarker detection in human serum. In addition, the storage stability of the immunoelectrodes was examined by determining 500 U mL^−1^ CA125 and 500 ng mL^−1^ IgG, respectively. Seven samples of each type of immunoelectrode were prepared in parallel, and the test was conducted by using one electrode every day for one week. The peak current signals all reach over 95% of the initial value, suggesting good storage stability of our immunoelectrodes (as shown in Figure S7).

**Figure 6:**
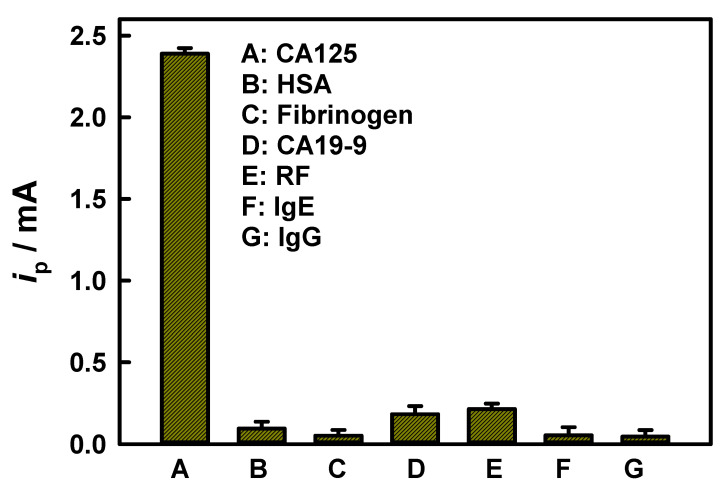
Selectivity of the CA125 immunoassay using our method. CA125 concentration is 50 mU mL^−1^, while concentration of CA19-9 is 50 U mL^−1^; RF and IgE are 50 IU mL^−1^; HAS, fibrinogen and IgG are 500 µg mL^−1^.

Moreover, the immunoassay’s resistance to interference toward CA125 (50 mU mL^−1^) was evaluated against several potential interferents at higher concentrations, including albumin (HAS, 500 μg mL^−1^), fibrinogen (500 μg mL^−1^), carcinoembryonic antigen 19-9 (CA19-9, 50 U mL^−1^), rheumatoid factors (RF, 50 IU mL^−1^), immunoglobulin E (IgE, 50 IU mL^−1^) and IgG (500 μg mL^−1^). The experimental conditions are the same as those mentioned above. It should be noted here that although the concentration units of the above interfering substances are somewhat different, the concentrations we measured are all extremely high values compared to the normal concentrations in the human body. The results demonstrated that only CA125 elicited a significantly increased current response (Figure 6). This highlights the exceptional selectivity of our method for CA125 detection, which is attributable primarily to the high specificity that is inherent to the anti-human CA125 antibody for its target.

## 4. Conclusions

We proposed a novel electrochemical method for immunoassays, on the basis of enzyme-labeled bionanocomposite and enzyme-catalyzed metal amplification, through the concurrent chemical dissolution/electrochemical-cathodic electrodeposition of gold, enabling in situ microliter sample detection via an ASV detection on the immunoelectrode. The results obtained from the electrochemical immunoassay of CA125 based on our method are consistent with the hospital results in clinical samples. The method offers high sensitivity, a wide linear detection range, and an exceptionally low LOD. Additional advantages include excellent precision, minimal reagent/sample consumption, and potential applicability in clinical diagnostics, point-of-care testing and biochemical analysis.

## Figures and Tables

**Table 2:** CA125 measurement results in human serum samples.

Serum Samples	Reference Method ^[a]^/U mL^−1^	Our Method ^[b]^/U mL^−1^	RSD(%)
1	57.3	54.6	−4.9
2	77.8	84.9	9.1
3	110	115	4.3
4	128	138	8.2
5	3513	3444	−2.0

[a] The chemiluminescence served as the reference method. [b] The data here represent the average value of three repeated tests.

## Data Availability

The original contributions presented in this study are included in the article. Further inquiries can be directed to the corresponding authors.

## Supplementary Materials

The following supporting information can be downloaded at: https://www.mdpi.com/article/10.3390/bios15100689/s1, Figure S1: UV-Vis spectra and digital picture (Insert) of 2 mM NaAuCl_4_ and 50 µM H_2_O_2_ for 0 h (A) and after reaction of 2 mM NaAuCl_4_ and 50 µM H_2_O_2_ for 2 h (B); Figure S2: Time-dependent QCM-frequency responses of the process of immune affinity reaction and enzyme-catalyzed gold deposition. The arrow represents the moment of addition; Figure S3: TEM image of PABA-AuNPs-GOx; Figure S4: EDX spectra of Ab_2_-PABA-AuNPs-GOx. It should be noted here that the copper element signal of EDX originates from the copper mesh substrate used during the TEM test process; Figure S5: Effects of glucose concentration (A), NaAuCl_4_ concentration (B) and enzymatic reaction time (C). Reaction solution medium: pH 7.4 0.01 M PBS. Concentration of IgG: 500 ng mL^−1^; Figure S6: Linear sweep ASV curves for IgG (A) or CA125 (B) detection by using different carriers of secondary antibody. Detection solutions: pH 7.4 PBS containing 50 ng mL^−1^ IgG and CA125 is 50 mU mL^−1^ CA125, respectively; Figure S7: Storage stability of the immunoelectrodes under storage conditions, curve a represents the detection of CA125, and curve b represents the detection of IgG. Detection solutions: pH 7.4 PBS containing 500 U mL^−1^ CA125 (curve a) and 500 ng mL^−1^ IgG (curve b), respectively. When not in use, the prepared immunoelectrodes were stored in PBS at 4 °C (refrigerator).

## References

[1] Huang R., He L., Jin L., Li Z., He N., Miao W. (2023). Recent advancements in DNA nanotechnology-enabled extracellular vesicles detection and diagnosis: A mini review. Chin Chem. Lett..

[2] Yang G., Li Z., Usman R., Chen Z., Liu Y., Li S., Chen H., Deng Y., Fang Y., He N. (2025). DNA walker induced “signal on” fluorescence aptasensor strategy for rapid and sensitive detection of extracellular vesicles in gastric cancer. Chin. Chem. Lett..

[3] Rafat N., Zhang H., Rudge J., Kim Y.N., Peddireddy S.P., Das N., Sarkar A. (2022). Enhanced enzymatically amplified metallization on nanostructured surfaces for multiplexed point-of-care electrical detection of COVID-19 biomarkers. Small.

[4] Fang Y., Wang Y., Zhu L., Liu H., Su X., Liu Y., Chen Z., Chen H., He N. (2023). A novel cartridge for nucleic acid extraction, amplification and detection of infectious disease pathogens with the help of magnetic nanoparticles. Chin. Chem. Lett..

[5] Zhao X., Gao J., Shi K., Zhang C., Ma W., Lyu G., Zhang J., Lu J., Liu Q., Luo X. (2025). A new radioactive microsphere: Y-90 carbon microsphere for selective internal radiation therapy of advanced liver cancer. Chin. Chem. Lett..

[6] Wu Y., Zhang Z., Wei Y., Qian Z., Wei X. (2023). Nanovaccines for cancer immunotherapy: Current knowledge and future perspectives. Chin. Chem. Lett..

[7] Puiu M., Nativi C., Bal C. (2023). Early detection of tumour-associated antigens: Assessment of point-of-care electrochemical immunoassays. TrAC-Trend. Anal. Chem..

[8] Ran H., Wang S., Ju F., Huang Z., Gao Y., Zhang J., He N., Nie L. (2025). Metal nanocluster-based biosensors for DNA detection. Biosensors.

[9] Huang Z., Wu K., Ju F., He R., Tang Y., Chen Y., He X., Zhang J., Nie L. (2025). Copper nanocluster based cascade amplified DNA electrochemical detection combining with bio-barcode assay and surface-initiated enzyme polymerization. Bioelectrochemistry.

[10] Tang Z., Ma Z. (2017). Multiple functional strategies for amplifying sensitivity of amperometric immunoassay for tumor markers: A review. Biosens. Bioelectron..

[11] Yang G., Lai Y., Xiao Z., Tang C., Deng Y. (2018). Ultrasensitive electrochemical immunosensor of carcinoembryonic antigen based on gold-label silver-stain signal amplification. Chin. Chem. Lett..

[12] Lai G., Yan F., Wu J., Leng C., Ju H. (2011). Ultrasensitive multiplexed immunoassay with electrochemical stripping analysis of silver nanoparticles catalytically deposited by gold nanoparticles and enzymatic reaction. Anal. Chem..

[13] Sui Y., Xu A., Jin X., Zheng J., He X., Cheng Y., Xie Q., Liu R. (2018). In situ enzymatic generation of gold for ultrasensitive amperometric sandwich immunoassay of procalcitonin. Biosens. Bioelectron..

[14] Ge S., Liang L., Lan F., Zhang Y., Wang Y., Yan M., Yu J. (2016). Photoelectrochemical immunoassay based on chemiluminescence as internal excited light source. Sens. Actuator B Chem..

[15] Liu D., Yang J., Wang H.-F., Wang Z., Huang X., Wang Z., Niu G., Walker A.R.H., Chen X. (2014). Glucose oxidase-catalyzed growth of gold nanoparticles enables quantitative detection of attomolar cancer biomarkers. Anal. Chem..

[16] Chen Z.-P., Peng Z.-F., Luo Y., Qu B., Jiang J.-H., Zhang X.-B., Shen G.-L., Yu R.-Q. (2007). Successively amplified electrochemical immunoassay based on biocatalytic deposition of silver nanoparticles and silver enhancement. Biosens. Bioelectron..

[17] Zhang J., Pearcea M.C., Ting B.P., Ying J.Y. (2011). Ultrasensitive electrochemical immunosensor employing glucose oxidase catalyzed deposition of gold nanoparticles for signal amplification. Biosens. Bioelectron..

[18] Hwang S., Kim E., Kwak J. (2005). Electrochemical detection of DNA hybridization using biometallization. Anal. Chem..

[19] Xu A., Chao L., Xiao H., Sui Y., Liu J., Xie Q., Yao S. (2018). Ultrasensitive electrochemical sensing of Hg^2+^ based on thymine-Hg^2+^-thymine interaction and signal amplification of alkaline phosphatase catalyzed silver deposition. Biosens. Bioelectron..

[20] Qin X., Xu A., Liu L., Deng W., Chen C., Tan Y., Fu Y., Xie Q., Yao S. (2015). Ultrasensitive electrochemical immunoassay of proteins based on in situ duple amplification of gold nanoparticle biolabel signals. Chem. Commun..

[21] Moreno-Guzmán M., Ojeda I., Villalonga R., González-Cortés A., Yáñez-Sedeño P., Pingarrón J.M. (2012). Ultrasensitive detection of adrenocorticotropin hormone (ACTH) using disposable phenylboronic-modified electrochemical immunosensors. Biosens. Bioelectron..

[22] Fu Y., Li P., Xie Q., Xu X., Lei L., Chen C., Zou C., Deng W., Yao S. (2009). One-pot preparation of polymer-enzyme-metallic nanoparticle composite films for high-performance biosensing of glucose and galactose. Adv. Funct. Mater..

[23] Bitinis N., Hernandez M., Verdejo R., Kenny J.M., Lopez-Manchado M.A. (2011). Recent advances in clay/polymer nanocomposites. Adv. Mater..

[24] Huang Y., Qin X., Li Z., Fu Y., Qin C., Wu F., Su Z., Ma M., Xie Q., Yao S. (2012). Fabrication of a chitosan/glucose oxidase–poly(anilineboronic acid)–Au_nano_/Au-plated Au electrode for biosensor and biofuel cell. Biosens. Bioelectron..

[25] Xie Q., Wang J., Zhou A., Zhang Y., Liu H., Xu Z., Yuan Y., Deng M., Yao S. (1999). A study of depletion layer effects on equivalent circuit parameters using an electrochemical quartz crystal impedance system. Anal. Chem..

[26] Fu Y., Li P., Bu L., Wang T., Xie Q., Xu X., Lei L., Zou C., Yao S. (2010). Chemical/biochemical preparation of new polymeric bionanocomposites with enzyme labels immobilized at high load and activity for high-performance electrochemical immunoassay. J. Phys. Chem. C.

[27] Annavarapu S., Apelian D., Lawley A. (1990). Spray casting of steel strip: Process analysis. Metall. Mater. Trans. A.

[28] Li Z., Pan J., Tang J. (2003). Highly sensitive and selective spectrophotometric method for determination of trace gold in geological samples with 5-(2-hydroxy-5-nitrophenylazo)rhodanine. Anal. Bioanal. Chem..

[29] Comamala M., Pinard M., Thériault C., Matte I., Albert A., Boivin M., Beaudin J., Piché A., Rancourt C. (2011). Downregulation of cell surface CA125/MUC16 induces epithelial-to-mesenchymal transition and restores EGFR signalling in NIH: OVCAR3 ovarian carcinoma cells. Brit. J. Cancer..

[30] Liu K., Zhang J.-J., Wang C., Zhu J.-J. (2011). Graphene-assisted dual amplification strategy for the fabrication of sensitive amperometric immunosensor. Biosens. Bioelectron..

[31] Chen M., Song Z., Han R., Li Y., Luo X. (2021). Low fouling electrochemical biosensors based on designed Y-shaped peptides with antifouling and recognizing branches for the detection of IgG in human serum. Biosens. Bioelectron..

[32] Cui R., Pan H.-C., Zhu J.-J., Chen H.-Y. (2007). Versatile immunosensor using CdTe quantum dots as electrochemical and fluorescent labels. Anal. Chem..

[33] Jofre C.F., Regiart M., Fernández-Baldo M.A., Bertotti M., Raba J., Messina G.A. (2020). Electrochemical microfluidic immunosensor based on TESAuNPs@Fe_3_O_4_ and CMK-8 for IgG anti-Toxocara canis determination. Anal. Chim. Acta.

[34] Qin X., Liu L., Xu A., Wang L., Tan Y., Chen C., Xie Q. (2016). Ultrasensitive immunoassay of proteins based on gold label/silver staining, galvanic replacement reaction enlargement, and in situ microliter-droplet anodic stripping voltammetry. J. Phys. Chem. C.

[35] Qin X., Xu A., Wang L., Liu L., Chao L., He F., Tan Y., Chen C., Xie Q. (2016). In situ microliter-droplet anodic stripping voltammetry of copper stained on the gold label after galvanic replacement reaction enlargement for ultrasensitive immunoassay of proteins. Biosens. Bioelectron..

[36] Wang J., Xie J., Chen M., Zheng S., Tan H., Yu S., Chen Y., Huang X., Gao W. (2023). Core-double-shell structure and heterojunction in CdS/SnS_2_ boosting fast carrier separation for ultrasensitive detection of carbohydrate antigen 125. Sens. Actuator B Chem..

[37] Guo A., Wu D., Ma H., Zhang Y., Li H., Du B., Wei Q. (2013). An ultrasensitive enzyme-free electrochemical immunosensor for CA125 using Au@Pd core–shell nanoparticles as labels and platforms for signal amplification. J. Mater. Chem. B.

[38] Liu Z., Huang Q., Yan Y., Yao J., Zhong F., Xie S., Zhang M., Zhang H., Jin M., Shui L. (2023). A multi-unit integrated electrochemical biosensor array for synergistic signal enhancing carbohydrate antigen 125 detection. Sens. Actuator B Chem..

[39] Raghav R., Srivastava S. (2015). Core-shell gold-silver nanoparticles based impedimetric immunosensor for cancer antigen CA125. Sens. Actuator B Chem..

[40] Tang M., Wen G., Luo Y., Liang A., Jiang Z. (2015). A simple resonance Rayleigh scattering method for determination of trace CA125 using immuno-AuRu nanoalloy as probe via ultrasonic irradiation. Spectrochim. Acta A.

[41] Al-Ogaidi I., Aguilar Z.P., Suri S., Gou H., Wu N. (2013). Dual detection of cancer biomarker CA125 using absorbance and electrochemical methods. Analyst.

[42] Tang D., Su B., Tang J., Ren J., Chen G. (2010). Nanoparticle-Based sandwich electrochemical immunoassay for carbohydrate antigen 125 with signal enhancement using enzyme-coated nanometer-sized enzyme-doped silica beads. Anal. Chem..

[43] Ge J., Dou J., Yu X., Sun Y., Song H., Shen D. (2024). Simultaneous determination of α-fetoprotein and carbohydrate antigens 125 and 19-9 in potential- and spectrum-resolved dual-mode electrochemiluminescence. Microchem. J..

