# Supplementary material for: Ultrasensitive Electrochemical Immunoassays of IgG and CA125 Based on Glucose Oxidase-Catalyzed Signal Amplification with Gold Staining
Source: Biosensors (Basel). 2025 Oct 11;15(10):689. doi: 10.3390/bios15100689 (PMC12564282; doi:10.3390/bios15100689)
Supplement: Supplementary file 1 [file biosensors-15-00689-s001.zip › biosensors-3849311-supplementary.pdf]

Supplementary Materials

Ultrasensitive Electrochemical Immunoassays of IgG and CA125 Based on Glucose Oxidase-Catalyzed Signal Amplification with Gold Staining

Long Chao [1,2,*], Zhisong Wu [1], Shiqiang Qi [1], Aigui Xu [2], Zhao Huang [1] and Dexuan Yan [1,3,*]

1 Hunan Key Laboratory of Biomedical Nanomaterials and Devices, School of Biological Sicence and Medical Engineering, Hunan University of Technology, Zhuzhou 412007, China
2 Key Laboratory of Chemical Biology and Traditional Chinese Medicine Research, Ministry of Education of China, College of Chemistry and Chemical Engineering, Hunan Normal University, Changsha 410081, China
3 College of Design and Engineering, National University of Singapore, 21 Lower Kent Ridge Road, Singapore 119077, Singapore
* Correspondence: chaolong4617@163.com (L.C.); yandxuan@163.com (D.Y.)

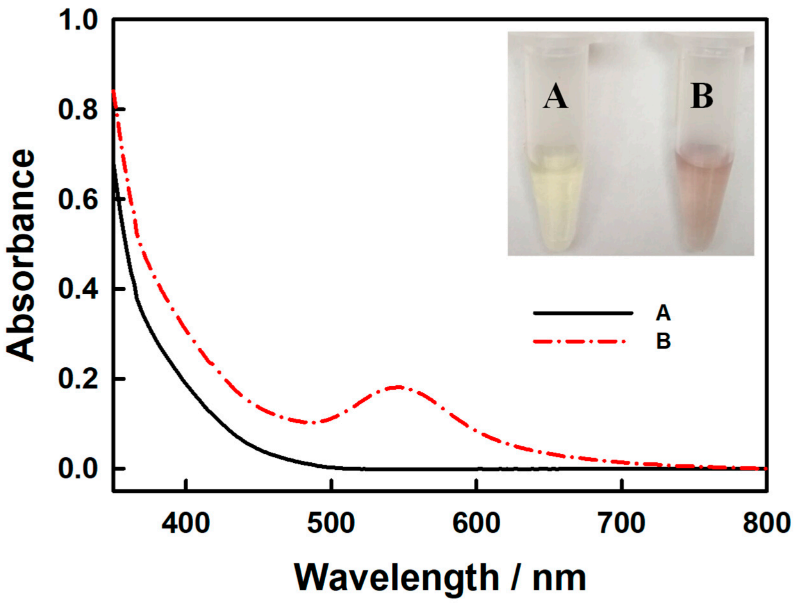

Figure S1. UV-Vis spectra and digital picture (Insert) of 2 mM $NaAuCl_4$ and 50 μM H_2O_2 for 0 h (A) and after reaction of 2 mM $NaAuCl_4$ and 50 μM H_2O_2 for 2 h (B).

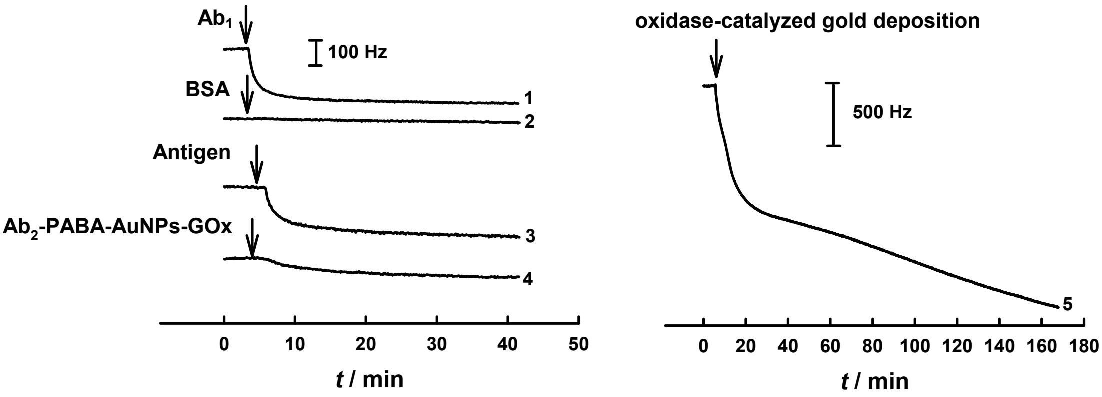

Figure S2. Time-dependent QCM-frequency responses of the process of immune affinity reaction and enzyme-catalyzed gold deposition. The arrow represents the moment of addition.

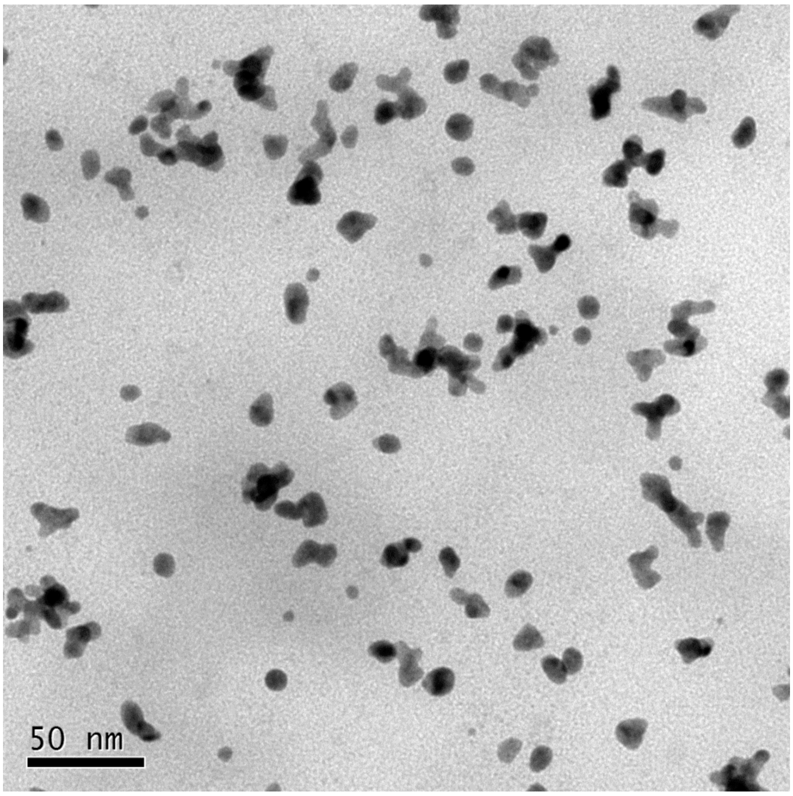

Figure S3. TEM image of PABA-AuNPs-GOx.

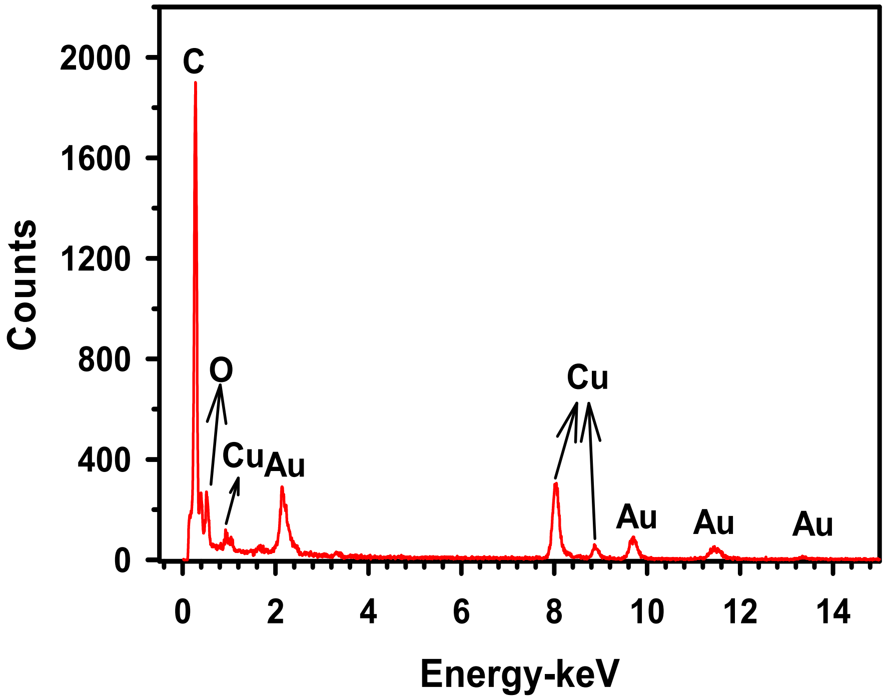

Figure S4. EDX spectra of Ab_2-PABA-AuNPs-GOx. It should be noted here that the copper element signal of EDX originates from the copper mesh substrate used during the TEM test process.

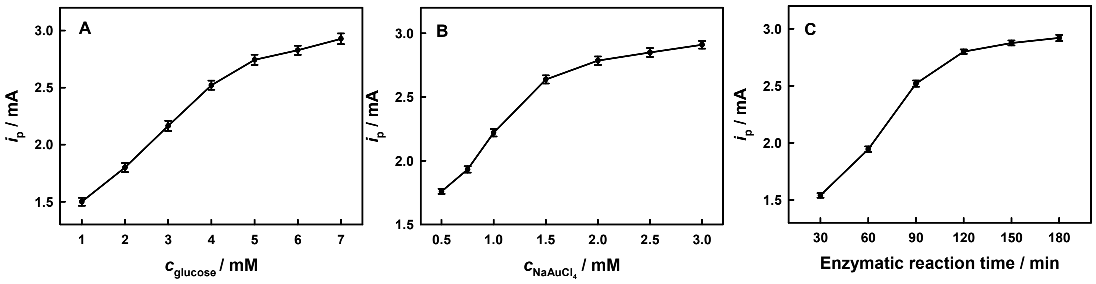

Figure S5. Effects of glucose concentration (A), $NaAuCl_4$ concentration (B) and enzymatic reaction time (C). Reaction solution medium: pH 7.4 0.01 M PBS .Concentration of IgG: 500 ng mL^{-1}.

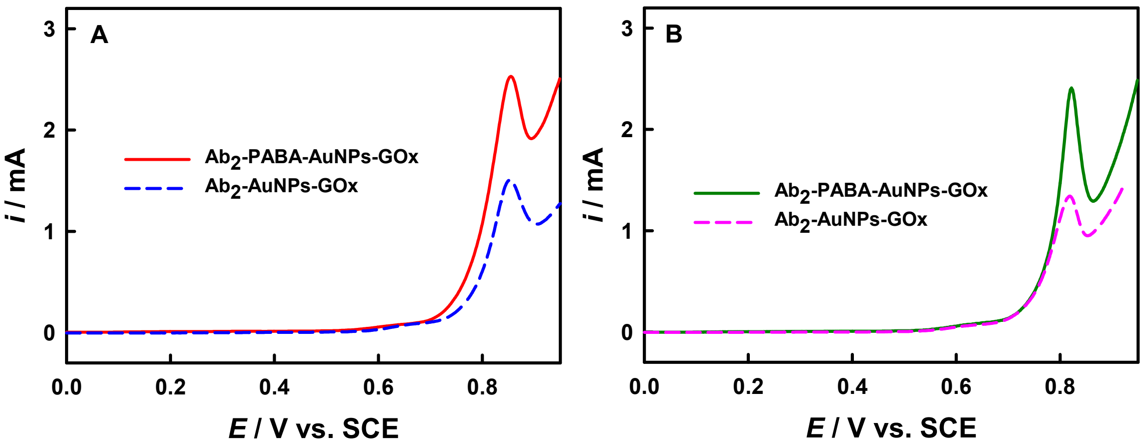

Figure S6. Linear sweep ASV curves for IgG (A) or CA125 (B) detection by using different carriers of secondary antibody. Detection solutions: pH 7.4 PBS containing 50 ng mL^{-1} IgG and CA125 is 50 mU mL^{-1} CA125, respectively.

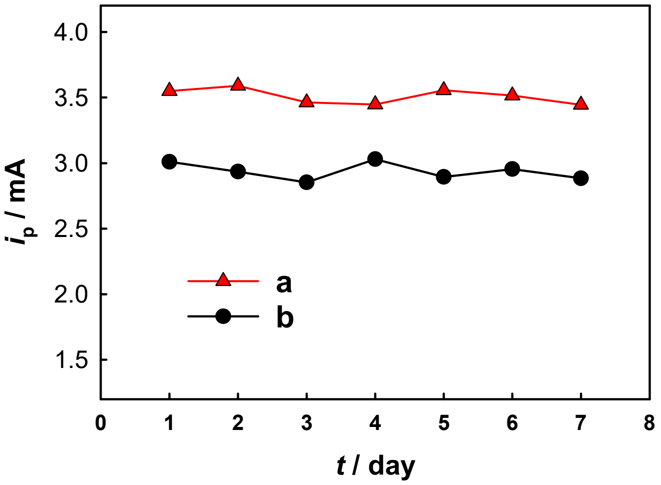

Figure S7. Storage stability of the immunoelectrodes under storage conditions, curve a represents the detection of CA125, and curve b represents the detection of IgG. Detection solutions: pH 7.4 PBS containing 500 U mL^{-1} CA125 (curve a) and 500 ng mL^{-1} IgG (curve b), respectively. When not in use, the prepared immunoelectrodes were stored in PBS at 4 °C (refrigerator).
